# Irinotecan in patients with relapsed or cisplatin-refractory germ cell cancer: a phase II study of the German Testicular Cancer Study Group

**DOI:** 10.1038/sj.bjc.66000524

**Published:** 2002-09-23

**Authors:** C Kollmannsberger, O Rick, H Klaproth, T Kubin, H G Sayer, M Hentrich, M Welslau, F Mayer, M Kuczyk, C Spott, L Kanz, C Bokemeyer

**Affiliations:** Department of Haematology/Oncology, University of Tuebingen Medical Center, Germany; Department of Haematology/Oncology, Charite, Berlin, Germany; Department of Haematology/Oncology, University of Goettingen, Germany; Department of Haematology/Oncology, Städt. Klinikum, Karlsruhe, Germany; Department of Haematology/Oncology, University of Jena, Germany; Department of Haematology/Oncology, Krankenhaus Harlaching, München, Germany; Onkologische Praxis Klausmann/Welslau, Aschaffenburg, Germany; Department of Urology, Hannover University Medical School, Germany

**Keywords:** germ cell cancer (GCT), irinotecan, cisplatin-refractory, relapse, palliative chemotherapy

## Abstract

Despite generally high cure rates in patients with metastatic germ cell cancer, patients with progressive disease on first-line cisplatin-based chemotherapy or with relapsed disease following high-dose salvage therapy exhibit a very poor prognosis. Irinotecan has shown antitumour activity in human testicular tumour xenografts in nude mice. We have performed a phase II study examining the single agent activity of irinotecan in patients with metastatic relapsed or cisplatin-refractory germ cell cancer. Refractory disease was defined as progression or relapse within 4 weeks after cisplatin-based chemotherapy or relapse after salvage high-dose chemotherapy with autologous stem cell support. Irinotecan was administered at a dose of 300 (−350) mg m^−2^ every 3 weeks. Response was evaluated every 4 weeks. Fifteen patients have been enrolled. Median age was 35 (19–53) years. Primary tumour localisation was gonadal/mediastinal in 12/3 patients. Patients had been pretreated with a median of six (4–12) cisplatin-containing cycles and 13 out of 15 patients had previously failed high-dose chemotherapy with blood stem cell support. Median number of irinotecan applications was two (1–3). Fourteen patients are assessable for response and all for toxicity. In one patient, no adequate response evaluation was performed. Toxicity was generally acceptable and consisted mainly of haematological side effects with common toxicity criteria 3° anaemia (two patients), common toxicity criteria 3° leukocytopenia (one patient) and common toxicity criteria 3° thrombocytopenia (three patients). Common toxicity criteria 3/4° non-haematological toxicity occurred in five patients (33%): 1×diarrhoea, 2×alopecia, 1×fever and in one patient worsening of pre-existing peripheral polyneuropathy from 1° to 4°. No response was observed to irinotecan therapy. Currently, 13 patients have died of the disease and two patients are alive with the disease. The patients included in our study exhibit similar prognostic characteristics as patients treated in previous trials evaluating new drugs in this setting. Irinotecan at a dose of 300–350 mg m^−2^ every 3 weeks appears to have no antitumour activity in patients with cisplatin-refractory germ cell cancer and, thus, further investigation in this disease is not justified.

*British Journal of Cancer* (2002) **87**, 729–732. doi:10.1038/sj.bjc.6600524 www.bjcancer.com

© 2002 Cancer Research UK

## 

Today, approximately 70–80% of patients with metastatic germ cell cancer can be cured with cisplatin-based combination chemotherapy (Einhorn, 1990; Bosl and Motzer, 1997). Patients who relapse after first-line chemotherapy will achieve long-term survival rates of only 20–50% following platinum-containing standard- or high-dose salvage chemotherapy. Patients progressing during or relapsing after salvage chemotherapy exhibit an extremely poor prognosis and long-term survival is achieved in less than 5% of patients (Nichols *et al*, 1994; Porcu *et al*, 2000). The identification of new drugs with significant antitumour activity remains a priority in these heavily pretreated patients. Several cytotoxic agents, including topotecan, vinorelbine, iproplatin, paclitaxel, and more recently, bendamustine, gemcitabine and oxaliplatin have been investigated in refractory germ cell cancer patients (Drasga *et al*, 1987; Murphy *et al*, 1992; Bokemeyer *et al*, 1993, 1996, 1999; Motzer *et al*, 1994; Puc *et al*, 1995; Einhorn *et al*, 1999; Kollmannsberger *et al*, 2000, 2001). However, only continuously applied low dose oral etoposide, paclitaxel, gemcitabine, and, most recently, oxaliplatin have thus far been able to demonstrate response rates of about 13–20% in patients with cisplatin-refractory germ cell cancer (Miller and Einhorn, 1990; Motzer *et al*, 1994; Bokemeyer *et al*, 1996, 1999; Einhorn *et al*, 1999; Kollmannsberger *et al*, 2001).

Irinotecan (CPT-11; 7-ethyl-10 hydroxycamptothecin 10(1,4′-biperidene)-carboxylat) is a topoisomerase-I inhibitor that blocks the DNA replication step of the enzyme, leading to multiple single-strand DNA breaks eventually blocking cell division (Creemers *et al*, 1994). No cross-resistance has thus far been reported to other agents active in germ cell cancer such as cisplatin, ifosfamide or bleomycin. Irinotecan has been approved for the first- and second-line treatment of metastatic colon cancer (Cunningham *et al*, 1998; Douillard *et al*, 2000). Common side effects of irinotecan include delayed diarrhoea, neutropenia, early cholinergic syndrome, and nausea/vomiting. The dose-limiting toxicities are severe diarrhoea and neutropenia. *In vitro* data demonstrated a dose-dependent activity of irinotecan against human testicular tumour xenografts (Miki *et al*, 1997). This provided the rationale for the present study which evaluates irinotecan in pretreated patients with refractory germ cell cancer.

## PATIENTS AND METHODS

Eligibility requirements included the diagnosis of germ cell cancer with evidence of tumour progression or relapse after at least two previous adequate cisplatin-based chemotherapies or after salvage high-dose chemotherapy with autologous stem cell support. Patients with disease progression during their initial induction chemotherapy or during salvage therapy were also eligible. Additional inclusion criteria were the presence of bidimensionally measurable disease and/or elevated tumour markers, a Karnofsky performance status ⩾60% as well as adequate haematological (WBC >2500 μl^−1^, platelets >75 000 μl^−1^), renal (creatinine clearance >60 ml min^−1^) and liver function (bilirubin ⩽1.5-fold upper normal value; liver enzymes <three-fold upper normal value). No other concomitant chemotherapy, radiotherapy or experimental medication was allowed. All patients had to give their written informed consent. The study was approved by the University of Tuebingen Ethics Committee.

Irinotecan was administered at a dose of 300 mg m^−2^ infused over 30 min on day one, repeated every 3 weeks. In case of CTC (common toxicity criteria) toxicity ⩽°1, a dose escalation to 350 mg m^−2^ was planned. Concomitant antiemetic therapy included 5-HT_3_-antagonists as well as dexamethasone. A dose reduction of 50% was planned in case of CTC °4 thrombocytopenia, granulocytopenia or neutropenic fever. No routine use of G-CSF was recommended, but G-CSF could be given on an individual basis in instances of severe neutropenia. A 25% and 50% dose reduction was planned in case of CTC °3 or °4 non-haematological toxicity, respectively. All patients were treated on an outpatient basis.

### Definitions

Disease was considered cisplatin-refractory, when at least tumour stabilisation or a remission was achieved, but tumour progression occurred again within 4 weeks of the last cisplatin-based chemotherapy. Disease was considered absolutely cisplatin-refractory, when tumour progression developed while receiving cisplatin-based therapy (Nichols *et al*, 1989; Beyer *et al*, 1996).

Response and toxicity were graded according to WHO and NCI-CTC (version 2.0) criteria, respectively (Miller *et al*, 1981). In addition, reduction of the size of a tumour lesion and normalisation of previously elevated tumour markers was considered a partial remission with tumour marker normalisation (PR−), whereas a reduction 50% in the sum of the perpendicular diameters of measurable disease plus a tumour marker decrease of >50% for at least 1 month, but without complete normalisation, was considered a marker positive partial remission (PR+). Serum tumour markers were determined every 3 weeks. Evaluation of measurable disease by radiographic means was performed every 4 weeks. All responses as well as the diagnosis of stable disease had to be re-confirmed after a 4-week interval. All patients were scheduled to receive at least two cycles of treatment. However, in case of a significant marker (50%) and/or radiological progression (25%) after one cycle, the treatment was stopped and the patient was classified as having progressive disease. In patients with tumour responses or disease stabilisation therapy was planned to be continued for at least two more cycles after achievement of the best response unless severe toxicity occurred.

### Statistical considerations

The sequential two-step statistical design of Gehan was used to determine the number of patients required (Gehan, 1961). A response rate of clinical interest of 20%, which is comparable to other active drugs in this therapeutic situation, was assumed. Based on a significance level of 0.05 and a power of 80%, initially 14 patients had to be included. If no responses were observed, the trial would be closed because if the true response rate was at least 20%, then the probability of obtaining no responses in 14 patients was less than 5%. In case of one response, the number of patients required for the second step was to be calculated based on the total number of responders within the first 14 patients.

Survival and follow-up time were calculated from the beginning of irinotecan therapy until the date of death or the date of last follow-up using the Kaplan–Meier test.

## RESULTS

Fifteen patients were entered into the study between November 2000 and September 2001. Patient characteristics are listed in Table 1

**Table 1:**
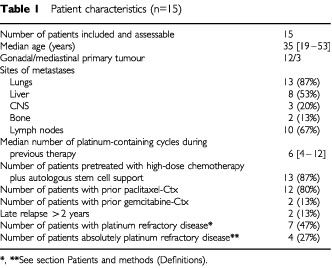
Patient characteristics (n=15)

. A total of 28 cycles of irinotecan were applied with a median of two cycles (range 1–3 cycles) per patient. Most patients presented with lung (87%) and lymph node (67%) metastases. Eight (53%) patients had liver involvement and three (20%) had brain metastases. Two (13%) patients had relapsed later than 2 years following initial therapy and 11 (73%) patients were considered to have platinum refractory or absolutely platinum refractory disease (Nichols *et al*, 1989; Beyer *et al*, 1996). All patients were heavily pretreated with a median number of six (range: 4–12) cisplatin-containing chemotherapy cycles prior to irinotecan therapy. Thirteen patients (87%) had previously received carboplatin/etoposide-based high-dose chemotherapy with autologous stem cell support. In addition 12 (80%) patients were pretreated with paclitaxel, six (40%) with oxaliplatin and two (13%) with gemcitabine.

No objective response was observed within our cohort of 15 relapsed or cisplatin refractory patients. All patients progressed during the first or second cycle of therapy. Irinotecan was well tolerated in this patient population (Table 2

**Table 2:**
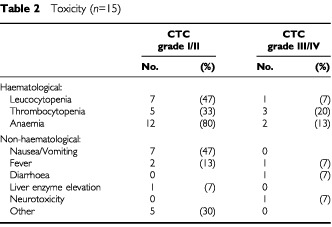
Toxicity (*n*=15)

). Five patients (30%) developed CTC °3/4 non-haematological side effects, one patient diarrhoea, two patients worsening of alopecia from °1 to °3 and °4, one patient a non-neutropenic infection and in one patient pre-existing polyneuropathy worsened from °1 to °4. Haematological toxicity was acceptable despite previous use of high-dose chemotherapy plus autologous stem cell support in almost all patients. Only three patients (20%) developed a CTC °3 thrombocytopenia and two patients (13%) CTC °3 anaemia. Forty-seven per cent of patients developed a CTC 1°/2° and one patient a °3 leucocytopenia. There were no cases of granulocytopenic fever or therapy-related mortality. After a median follow-up of 3 months (range 1–9 months) 13 patients (87%) have died and two patients (13%) are alive with disease. Median overall survival for all patients was 3 months (range 1–9).

## DISCUSSION

In recent years several new drugs have been systematically investigated for the treatment of patients with cisplatin-refractory testicular cancer. This exploration of new drugs has mostly involved intensively pretreated patients without established treatment options. The identification of new active agents in this setting, however, may not only allow palliative therapy for refractory patients but may also offer new possibilities for the development of combination therapy. Only very few of the cytostatic agents studied so far, in particular etoposide, ifosfamide or more recently paclitaxel have been found active in patients with refractory germ cell cancer and were subsequently incorporated into combination regimens. Due to its activity in refractory patients, paclitaxel was investigated as part of combination chemotherapy regimens in the salvage setting and is now studied in combination with the standard-PEB regimen as first-line treatment (Motzer *et al*, 2000) Two further agents, gemcitabine and oxaliplatin, have been reported to yield response rates of 13–19% in this patient population (Bokemeyer *et al*, 1999; Einhorn *et al*, 1999; Kollmannsberger *et al*, 2001).

The present phase II study investigates the activity of irinotecan in patients with intensively pretreated or cisplatin-refractory germ cell cancer. The rationale for the evaluation of irinotecan in testis cancer was based on the dose-dependent antitumour effect of irinotecan observed in human testicular tumour xenografts in nude mice either applied as single agent or in combination with cisplatin (Miki *et al*, 1997). A subsequent phase II study by Nomoto *et al* (2000) reported a very high response rate of 57% for the combination of irinotecan and cisplatin or nedaplatin in 14 cisplatin-refractory germ cell cancer patients suggesting that irinotecan may also be clinically active in refractory patients. No full report of the study by Nomoto *et al* (2000) has been published thus far and no data are available about the previous chemotherapy treatment, the response rate to previous treatment or the definition of ‘cisplatin-refractory disease’, which makes the correct interpretation of these results impossible. However, in our phase II study, no objective remission was observed in a cohort of 15 patients indicating that single agent irinotecan has no clinical antitumour activity in relapsed or cisplatin-refractory patients. Based on the negative results of our study, we assume that primarily cisplatin contributed to the response rate achieved in the Japanese study (Nomoto *et al*, 2000).

The patients in our study exhibited very unfavourable prognostic characteristics which may have prevented the activity of irinotecan. However, the patient population studied here was comparable to those who had been included in previous studies by our group investigating paclitaxel, gemcitabine or oxaliplatin in the refractory setting (Bokemeyer *et al*, 1996, 1999; Kollmannsberger *et al*, 2001). Considering the supposed preclinically dose-dependent efficacy of irinotecan, a very low dose of irinotecan might lead to negative clinical results. In our study, we administered irinotecan at a dose of 300 mg m^−2^ every 3 weeks with the plan to intraindividually increase the dose to 350 mg m^−2^ in case of good tolerability (⩽CTC °1 toxicity). This dose is very close to the officially approved single agent dose of irinotecan of 350 mg m^−2^, which has also been the maximally tolerated dose proposed from phase I studies. The starting dose of 300 mg/m^−2^ was chosen in order to prevent severe myelosuppression since nearly all patients were heavily pretreated including high-dose chemotherapy with autologous stem cell support. In addition, most patients developed at least °1 or °2 haematological toxicity indicating that the irinotecan dose was adequate for this patient population in our study and under-dosing may not be a likely explanation for the lack of activity of irinotecan in our study.

Irinotecan is the second topoisomerase I inhibitor which was evaluated in cisplatin-refractory germ cell cancer based on favourable preclinical data. Topotecan has also been shown to possess no clinical activity in this therapeutic setting (Puc *et al*, 1995). Thus, in contrast to topoisomerase II inhibitors, particularly etoposide, topoisomerase I inhibitors appear to be ineffective and this therapeutic principle seems to play no role in the treatment of germ cell cancer.

The good tolerability of irinotecan in this heavily pretreated group of patients was, however, remarkable. Haematological toxicity was acceptable, even in patients who had previously received high-dose chemotherapy plus autologous stem cell support. The treatment of patients, who may have a limited bone marrow function and thus are likely to develop an increased haematological toxicity, still remains a therapeutic challenge. Only few agents have been systematically investigated in this respect and were found to be feasible in this setting, among them paclitaxel, gemcitabine and bendamustine (Bokemeyer *et al*, 1996; Bregni *et al*, 1998; Constenla *et al*, 1998; Sola *et al*, 1999; Kollmannsberger *et al*, 2000). In all of them, thrombocytopenia appears to be more frequent than granulocytopenia. Thus, irinotecan's acceptable toxicity profile makes it a potential therapeutic option for the palliative treatment of heavily pretreated patients with cancer types for which a higher level of antitumour activity can be expected (Rosen, 1998; Rocha-Lima *et al*, 2001; Takeda *et al*, 2001; Ueoka *et al*, 2001). The further evaluation of irinotecan in patients with germ cell cancer is not recommended.
